# Associations of VICTORION-1 PREVENT eligibility with subclinical cardiovascular and inflammatory damage in a European population-based cohort

**DOI:** 10.1016/j.ajpc.2026.101524

**Published:** 2026-04-23

**Authors:** Nelson Wang, Deepak L. Bhatt, Constance Xhaard, Rahul Aggarwal, Marc P. Bonaca, Anastasia Lesogor, Janna Sand Dejmek, Manesh R. Patel, Erik S.G. Stroes, Pam R. Taub, Stephan Windecker, Kevin Duarte, Luca Monzo, Guillaume Baudry, Faiez Zannad, Nicolas Girerd, on behalf of the V-1P steering committee and STANISLAS study group

**Affiliations:** a The George Institute for Global Health, UNSW, Sydney, Australia; b Brigham and Women’s Hospital and Harvard Medical School, Boston, MA, USA; c Mount Sinai Fuster Heart Hospital, Icahn School of Medicine at Mount Sinai, NY, NY, USA; d CHRU Nancy, Université de Lorraine, INSERM, Centre d’Investigation Clinique Plurithématique 1433 and Inserm U1116, F-CRIN INI-CRCT (Cardiovascular and Renal Clinical Trialists), Nancy, France; e Brigham and Women’s Hospital Heart and Vascular Center, Harvard Medical School, Boston, MA, USA; f Division of Cardiology, Department of Medicine, University of Colorado School of Medicine, Aurora, CO, USA; g Novartis Pharma AG, Basel, Switzerland; h Duke Clinical Research Institute and Division of Cardiology, Duke University, Durham, NC, USA; i Department of Vascular Medicine, Amsterdam University Medical Center, Amsterdam, , Netherlands; j Department of Cardiovascular Medicine, University of California San Diego School of Medicine, La Jolla, CA, USA; k Department of Cardiology, Bern University Hospital, Inselspital, University of Bern, Freiburgstrasse, CH, 3010 Bern, Switzerland

**Keywords:** Primary prevention, Subclinical disease, Cholesterol

## Abstract

**Background and aims:**

The VICTORION-1 PREVENT (V-1P) trial is evaluating the efficacy of inclisiran versus placebo on cardiovascular events in primary prevention patients at high-risk for ASCVD. We assessed whether V-1P eligibility, based on Pooled Cohort Equations (PCE) and Predicting Risk of Cardiovascular Disease Events (PREVENT) equations, was associated with subclinical cardiovascular and inflammatory abnormalities in a healthy European population.

**Methods:**

We included individuals from the STANISLAS cohort in France aged 40–79 years, LDL-C 70–189 mg/dL and without ASCVD or liver disease. Participants were categorized as V-1P eligible using 10-year ASCVD risk using PCE and PREVENT. Associations with vascular, echocardiographic, and biomarkers were assessed using age- and sex-adjusted linear regression.

**Results:**

Among 848 participants (mean age 60 years, 51% female), 16% were eligible per PCE, of which 7% were also eligible with PREVENT. Only one participant was eligible by PREVENT alone. Compared with non-eligible participants, V-1P-eligible individuals, whether by PCE alone or PCE and PREVENT, displayed significant subclinical abnormalities. Compared with V-1P ineligible participants, V-1P eligible participants had increased intima media thickness (+51 µm, *p* < 0.009 for PCE+PREVENT) and increased mean pulse wave velocity (+0.89 m/s, *p* < 0.001 for both PCE and PCE+PREVENT) on vascular ultrasound. V-1P eligible participants by PCE+PREVENT also showed signs of subclinical myocardial injury and inflammation, with a 1.3 fold higher troponin (*p* = 0.015), 1.6-fold higher interleukin-6 (*p* < 0.001) and a 2-fold higher high sensitivity C-reactive protein (*p* < 0.001).

**Conclusions:**

A large proportion of asymptomatic individuals without known cardiovascular disease would be eligible for the V-1P trial based on both PCE and PREVENT equations. V-1P eligible participants had evidence of subclinical cardiovascular and inflammatory abnormalities.

**Figure:**
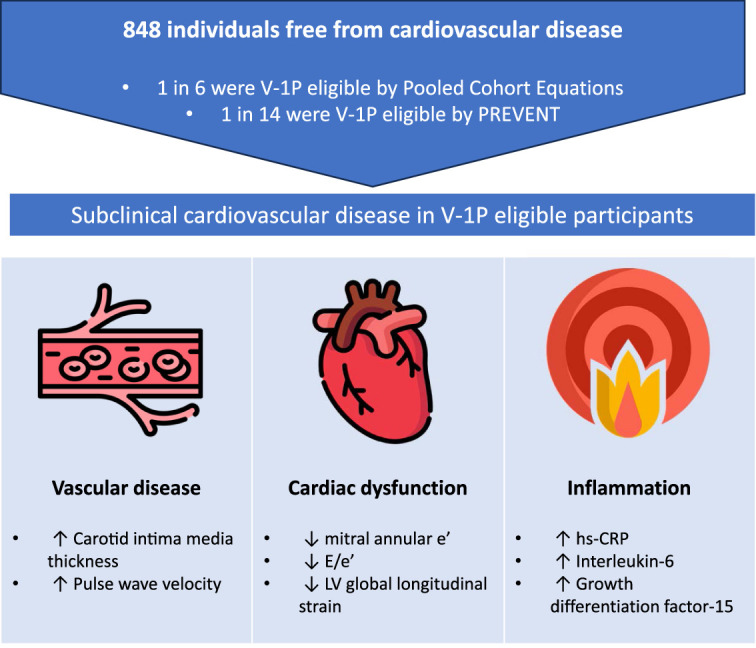
**Central Illustration.** Association between V-1P eligibility and subclinical cardiovascular disease in the STANISLAS cohort hs-CRP, high sensitivity C-reactive protein; LV, left ventricular; PCE, pooled cohort equation; PREVENT, Predicting Risk of Cardiovascular Disease Events.

## 1 Introduction

Cardiovascular disease (CVD) is the leading cause of death and disability globally [1]. Evidence from genetic, observational, and clinical trial data confirms that the cumulative burden of low-density lipoprotein cholesterol (LDL-C) exposure is a principal determinant of atherosclerotic cardiovascular disease (ASCVD) risk [2]. There has been growing interest in the role of LDL-C lowering earlier in the ASCVD disease process, which if sustained, will achieve substantially greater reductions in ASCVD over years or decades [[3], [4], [5]].

Inclisiran is a small-interfering RNA (siRNA) that suppresses hepatic proprotein convertase subtilisin/kexin type 9 (PCSK9) production and has been shown to reduce LDL-C by ∼50 % [6]. The role of inclisiran for primary prevention is of particular interest because of its six-monthly injection frequency and favourable efficacy and safety profile [7,8]. As with any pharmacological treatment decisions, benefits need to be weighed against risks and costs of treatment, particularly in a primary prevention cohort who are otherwise free from apparent cardiovascular disease. The VICTORION-1 PREVENT (V-1P) trial (NCT05739383) is testing inclisiran in a high-risk primary prevention population, defined as those with either evidence of non-obstructive coronary artery disease on computer tomography or invasive coronary angiogram, an elevated coronary artery calcium score or high predicted ASCVD risk (≥20 % over 10 years or 7.5–20 % with at least 2 risk enhancing factors) with an LDL-C between 70–190 mg/dL. However, it is unknown whether people who qualify based on predicted ASCVD risk will also show evidence of subclinical cardiovascular disease.

A prior study in the United States (US) identified that 17 % of adults aged 40–79 years of age were eligible for V-1P, and these individuals had high estimated 10-year ASCVD risk with low rates of statin use [9]. However, no such estimates have been performed in a European setting and no study has assessed for evidence of subclinical cardiovascular disease in high risk individuals otherwise free from established cardiovascular disease. The identification of subclinical disease in a primary prevention setting may support LDL-C lowering as well as targeted efforts for cardiovascular disease prevention.

Therefore, this study aimed to compare the proportion of participants who meet V-1P eligibility by the Pooled Cohort Equation (PCE) and Predicting Risk of Cardiovascular Disease Events (PREVENT) equations in a European cohort free from established cardiovascular and assess for evidence of subclinical cardiovascular disease and increased pro-inflammatory markers.

## 2 Methods

### 2.1 Study population

The STANISLAS (Suivi Temporaire Annuel Non‐Invasif de la Santé des Lorrains Assuré Sociaux) cohort is a single‐centre familial longitudinal cohort which includes 4295 participants (1006 families) from the Nancy region of France, who were first recruited at the Center for Preventive Medicine between 1993 and 1995 [10]. During 2011–2016, 1705 participants of the original 4295 participants participated in a fourth (STANISLAS‐V4) visit that consisted of detailed phenotyping including medical examination, an interview by trained nurses using a structured questionnaire, blood tests, echocardiography and vascular ultrasound [11]. The participants were initially healthy; however, a sizeable proportion of the cohort developed cardiovascular risk factors during follow-up and some developed cardiovascular disease. The local ethics committee (Comité de Protection des Personnes Est III, Nancy, France) approved the study protocol and all study participants provided written informed consent in order to participate.

**Fig. 1:**
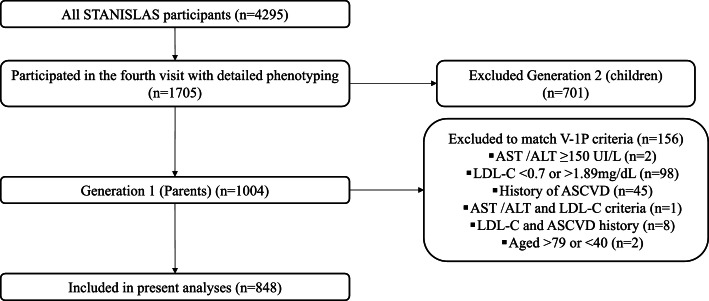
Study flow chart. ALT, alanine aminotransferase; ASCVD, atherosclerotic cardiovascular disease; AST, aspartate aminotransferase; LDL-C, low density lipoprotein cholesterol; V-1P, VICTORION-1 PREVENT.

For this study, we included STANISLAS-V4 participants who were aged 40–79 years and met V-1P trial eligibility criteria with an LDL-C of 70–189 mg/dL and were free from both ASCVD (defined as coronary artery disease, angina, myocardial infarction, or stroke) and liver disease (defined as alanine aminotransferase or aspartate aminotransferase ≥150 U/L) (Fig. 1). We calculated the 10-year ASCVD risk using both PCE [12] and the PREVENT ASCVD risk equations [13]. Participants met V-1P eligibility if the estimated 10-year ASCVD risk was ≥20 % or 7.5 %− 19.9 % with two cardiovascular risk enhancers. Risk enhancers, as defined in the V-1P trial, included elevated high-sensitivity C-reactive protein (hs-CRP) (hs-CRP, ≥2 mg/L), inflammatory conditions (psoriatic or rheumatoid arthritis), low estimated glomerular filtration rate (eGFR, <60), metabolic syndrome, or family history of a heart attack. LDL-C estimates were based on the Friedewald equation [14].

### 2.2 Vascular ultrasound

The protocol for the performance of vascular ultrasound in STANISLAS has previously been described [10]. High‐resolution echotracking in STANISLAS visit 4 was performed to assess both carotid plaques, diameter, distention, and cIMT on the right common carotid artery. Carotid to femoral pulse wave velocity (PWV) was measured in 825 (97.3 %) participants in accordance with the recommendations of the European Network for Non‐invasive Investigation of Large Arteries with Complior® (Alam Medical, France) and Sphygmocor® CVMS (AtCor, Australia) devices [15]. The mean value of two measurements was used to calculate the PWV. A third measurement was performed in cases when two measurements showed a difference of >0.5 m/s. The mean value of the three measurements was then used. Carotid intima-media thickness was measured in 833 (98.2 %) participants by high-resolution echo tracking on the right common carotid artery.

### 2.3 Two-dimensional standard echocardiography

Echocardiography was performed in 842 (99.3 %) of participants with a commercially available standard ultrasound scanner (Vivid 9, General Electric Medical Systems, Horten, Norway) 6 using a 2.5 MHz phased-array transducer (M5S). All echocardiographic and Doppler images were recorded in digital raw-data format and, after centralized anonymization, were submitted to the central core laboratory of the Centre d’Investigation Clinique (University of Lorraine, Nancy). All strain measurements were performed offline by two experienced echocardiographers with dedicated automated software (Q analysis software, Echo PAC PC version 110.1.0, GE Healthcare). Reproducibility analyses showed that inter- and intra-observer reproducibility of global longitudinal strain was good, with intra-class correlations >0.70 for all considered parameters for longitudinal strains[16,17].

### 2.4 Biomarkers measurements

Plasma samples were collected at the CIC-P de Nancy with minimally traumatic venipuncture. Standardized sample handling procedures enabled the collection of serum and plasma (EDTA, heparin) as well as buffy coat fraction. Circulating biomarkers (NT-proBNP, troponin, interleukin-6, growth differentiation factor-15) were analysed with the Olink Proseek®, using a proximity extension assay (PEA) technology. Growth differentiation factor 15 is associated with impaired myocardial energetics and function and predicts occurrence of coronary artery disease and incident heart failure [18,19]. All measured biomarkers are expressed as log2-normalized protein expression (NPX) data. Further details about the analytic process are described by Olink (https://www.olink.com/resources-support/white-papers-from-olink/).

### 2.5 Statistical analysis

First we calculated the 10-year estimated ASCVD risk using PCE and PREVENT [12,13]. Participants were then categorized according to three groups based on their trial eligibility according to estimated 10-year ASCVD risk: V-1P ineligible, V-1P eligible by PCE only (PCE group) and V-1P eligible by both PCE and PREVENT (PCE+PREVENT group). Only one participant was V-1P eligible by PREVENT but not PCE and was not included in grouped analyses. Data were reported using mean ± standard deviation (SD) or median and interquartile range. Comparisons between the three groups were performed using ANOVA test or Kruskal-Wallis test for continuous variables, as appropriate, and chi-square test or Fisher’s exact test for categorical variables. Linear regression analyses were used to assess the association between V-1P eligibility with vascular indices, echocardiographic variables and cardiac and inflammatory biomarkers. The echocardiographic measurements included for assessment were indexed LV mass, indexed LA volume, LA reservoir strain, LV global longitudinal strain, LV ejection fraction (LVEF), indexed LV end systolic volume, indexed LV end diastolic volume, average mitral annulus e’ velocity, mitral inflow velocity E and the ratio of mitral inflow velocity and mitral annular e' velocity (E/e'). Biomarker data derived from Olink proteomics were log2-transformed prior to analysis; thus, a one-unit increase in the regression coefficient corresponds to a doubling of the biomarker concentration. For each variable, both unadjusted and age- and sex-adjusted models were developed. A p-value <0.05 was considered statistically significant. All analyses were done with Stata version 18.1 and R version 4.1.2 [16,17].

## 3 Results

**Fig. 2:**
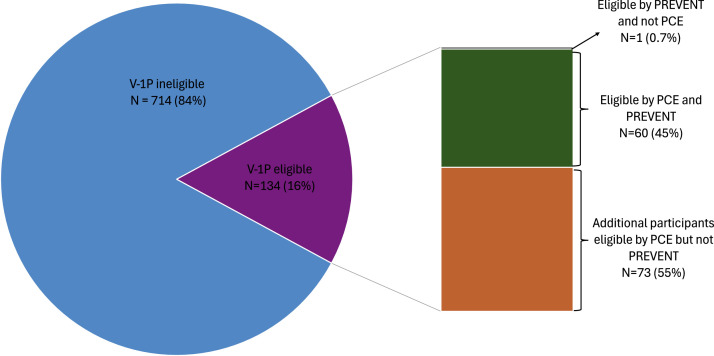
V-1P eligibility according to PCE and PREVENT equations. PCE pooled cohort equations; PREVENT, Predicting Risk of Cardiovascular Disease Events; V-1P, VICTORION-1 PREVENT.

Among 4295 participants in the STANISLAS cohort, 1004 adults had detailed phenotyping, of whom 156 were excluded to match the V-1P criteria (Fig. 1). Among the remaining 848 participants (mean age 60 ± 5 years, 51 % female), a total of 133 (15.7 %) met V-1P eligibility criteria by PCE, of whom 60 (7.1 %) were also eligible by PREVENT. Only one participant was eligible by PREVENT alone (Fig. 2, Table 1).

**Table 1:** Characteristics of participants according to V-1P eligibility.

Characteristics, units	V-1P ineligible		V-1P eligible based on PCE only		V-1P eligible based on PCE & Prevent		P-Value
	(*n* = 714)		(*n* = 73)		(*n* = 60)		
	n/mean	%/sd	n/mean	%/sd	n/mean	%/sd	
Mean Age, Years	59	(4)	63	(5)	65	(4)	<0.001
Women, n ( %)	407	(57)	10	(14)	16	(27)	<0.001
Mean Body mass index, kg/m^2^	26	(5)	29	(5)	32	(5)	<0.001
Mean systolic blood pressure, mmHg	127	(15)	141	(14)	146	(20)	<0.001
Diabetes, n ( %)	26	(4)	12	(16)	20	(33)	<0.001
Mean HbA1c, %	5.7	(0.4)	6.0	(0.9)	6.4	(1.1)	<0.001
Mean Fasting Blood Glucose, mg/dL	92	(12)	102	(24)	115	(32)	<0.001
Metabolic Syndrome, n ( %)	177	(25)	57	(78)	59	(98)	<0.001
Current smokers, n ( %)	68	(10)	18	(25)	8	(13)	<0.001
Mean Total Cholesterol, mg/dL	222	(30)	213	(32)	202	(34)	<0.001
Mean LDL-C, mg/dL	139	(26)	135	(29)	126	(29)	0.001
Mean HDL-C, mg/dL	62	(14)	53	(11)	47	(10)	<0.001
Mean Triglycerides, mg/dL	105	(49)	129	(65)	(147)	(66)	<0.001
Statin Use, n ( %)^a^	122	(17)	22	(30)	21	(35)	<0.001
Mean eGFR, ml/min/ 1.73 m2	89	(11)	87	(11)	86	(13)	0.12
Mean 10 Year ASCVD risk by PCE, %	6.4	(4.6)	17.1	(7.9)	21.5	(9.6)	<0.001
Mean 10 Year ACSVD risk by PREVENT, %	3.7	(2.0)	7.8	(3.4)	11.0	(4.2)	<0.001

ASCVD, atherosclerotic cardiovascular disease; eGFR, estimated glomerular filtration rate; HbA1c, glycated hemoglobin A1c; HDL-C, high density lipoprotein cholesterol; LDL-C, low density lipoprotein cholesterol; V-1P, VICTORION-1 PREVENT. a, three subjects were taking ezetimibe at baseline, all were V-1P ineligible.

Compared with ineligible participants, V-1P-eligible individuals were older (PCE+PREVENT: 65 ± 4 years, PCE-only: 63 ± 5 vs Ineligible: 59 ± 4, *p* < 0.001), had higher body mass index (32 ± 5, 29 ± 5 and 26 ± 5 kg/m², *p* < 0.001), and higher systolic blood pressure (146 ± 20, 141 ± 14 and 127 ± 15 mmHg, *p* < 0.001). A greater proportion of V-1P eligible participants were men (PCE+PREVENT 73 %, PCE only 86 % and ineligible 43 %), had diabetes (PCE+PREVENT 33 %, PCE only 16 % and ineligible 4 %), metabolic syndrome (PCE+PREVENT 98 %, PCE only 78 % and ineligible 25 %) and were current smokers (PCE+PREVENT 13 %, PCE only 25 % and ineligible 10 %) (all *p* < 0.001). Compared to participants who were eligible by PCE only, V-1P eligible participants by PCE+PREVENT were more often women, higher systolic blood pressure, more diabetes and more metabolic syndrome. Approximately 1 in 3 V-1P eligible participants were taking statins compared with 1 in 6 in V-1P ineligible participants (*p* < 0.001).

### 3.1 Estimated ASCVD risk

For all three groups, the estimated 10-year ASCVD risk was higher using PCE compared with PREVENT. The mean PCE 10-year ASCVD risk was 21 ± 10 % (PCE+PREVENT) and 17 ± 8 % (PCE only) for V-1P eligible participants compared to 6 ± 5 % for V-1P ineligible participants. The mean PREVENT 10-year ASCVD risk was 11 ± 4 % (PCE+PREVENT) and 8 ± 3 % (PCE only) for V-1P eligible participants compared to 4 ± 2 % for V-1P ineligible participants.

### 3.2 Biomarkers according to PCE/PREVENT eligibility

V-1P eligible participants also had higher levels of cardiac and inflammatory biomarkers compared to non-eligible participants (Table 2). For example, serum troponin for V-1P eligible participants was 0.9 ± 1.1 arbitrary units for PCE only and 1.2 ± 1.6 arbitrary units for PCE+PREVENT compared with 0.6 ± 1.0 V-1P ineligible participants (*p* < 0.001). Hs-CRP was 1.4 (0.8, 2.8) mg/L in V-1P ineligible participants, 2.4 (1.4, 3.9) in the PCE only group, and 3.7 (2.4, 6.5) in the PCE+PREVENT group (*p* < 0.001). Similarly, interleukin-6 and growth differentiation factor-15 were higher in V-1P eligible participants than ineligible participants (*p* < 0.001). Compared to participants who were V-1P eligible by PCE only, PCE+PREVENT participants exhibited greater abnormalities in biomarkers, including hs-CRP (2.4 [1.4; 3.9] vs 3.7 [2.4; 6.5]), NT-proBNP (3.8 ± 1.1 vs 4.2 ± 1.2), and interleukin-6 (3.3 ± 0.7 vs 3.8 ± 1.0).

**Table 2:** Vascular, echocardiographic, and biological variables according to V-1P eligibility.

	V-1P ineligible Population (40 - 79 years)		V-1P eligible population (PCE only)		V-1P eligible population (PCE & Prevent)		P-Value
	*n* = 714		*n* = 73		*n* = 60		
	Mean	SD, IQR or %	Mean	SD, IQR or %	Mean	SD, IQR or %	
**Vascular variables**							
Intima media thickness (µm)	697	135	738	152	782	146	<0.001
Pulse wave velocity (m/s)	8.9	1.6	10.4	2.4	10.5	1.6	<0.001
Carotid artery plaques, n ( %)	118	17	23	32	17	30	<0.001
**Echocardiographic variables**							
Indexed Left Ventricular Mass (g/m²)	79	19	86	19	87	25	<0.001
Indexed Left Atrial Volume (mL/m²)	23	8	24	8	24	10	0.48
LV Global longitudinal Strain	−21	3	−20	3	−18	4	<0.001
LV ejection fraction %	66	6	66	6	65	7	0.62
Indexed LV end-diastolic volume	48	13	48	13	47	13	0.68
Left Atrial Reservoir Strain %	30	7	30	8	27	7	0.10
E’ mean, cm/s	9.8	2.0	8.6	1.8	8.5	1.8	<0.001
E/e’ ratio	6.9	1.7	7.5	2.0	8.3	2.3	<0.001
E wave, m/s	0.7	0.1	0.6	0.2	0.7	0.1	0.17
A wave, m/s	0.67	0.15	0.74	0.20	0.78	0.15	<0.001
Tricuspid annular plane systolic excursion, mm	26	4	26	5	26	5	0.74
**Biomarker variables**							
NT-proBNP^a^	3.8	0.9	3.8	1.1	4.2	1.2	0.032
Troponin I^a^	0.6	1.0	0.9	1.1	1.2	1.6	<0.001
Interleukin-6^a^	3.0	0.7	3.3	0.7	3.8	1.0	<0.001
Growth differentiation factor-15^a^	6.0	0.4	6.3	0.4	6.5	0.6	<0.001
hs-CRP, mg/L	1.4	(0.8, 2.8)	2.4	(1.4, 3.9)	3.7	(2.4, 6.5)	<0.001

a, arbitrary units from Olink measured biomarkers; hs-CRP, High Sensitivity C-Reactive Protein; LV, left ventricular; V-1P, VICTORION-1 PREVENT.

### 3.3 Vascular and cardiac structure/function according to PCE/PREVENT eligibility

Vascular ultrasounds identified significant differences in the proportion of individuals who had evidence of carotid plaque(s) in V-1P eligible (32 % for PCE and 30 % for PCE+PREVENT) vs V-1P ineligible (17 %) participants (Table 2). There was also thicker carotid intima media and higher pulse wave velocity in V-1P eligible than V-1P ineligible participants. The intima media thickness was 697 ± 135 µm in ineligible participants, 738 ± 152 µm in PCE only group and 782 ± 146 µm in PCE+PREVENT group (*p* < 0.001). V-1P eligible participants had greater impairments in cardiac structure and function compared to V-1P ineligible patients with larger LV mass, more impaired LV global longitudinal strain, average mitral annular e' and E/e' (all *p* < 0.001).

Linear regressions comparing V-1P eligible to ineligible participants found significant differences in subclinical cardiovascular disease for several markers after adjusting for age and sex (Table 3). PCE+PREVENT eligible participants had significantly greater carotid intima media thickness than ineligible participants (*p* = 0.009), whilst both PCE only and PCE+PREVENT cohorts had greater pulse wave velocity than V-1P ineligible participants (both *p* < 0.001). For echocardiographic parameters, PCE+PREVENT eligible participants had more impaired LV global longitudinal strain compared to V-1P ineligible participants (*p* < 0.001). PCE only and PCE+PREVENT eligible participants also had more impaired e' and E/e' than ineligible participants. PCE+PREVENT eligible participants had higher troponin levels than ineligible participants (*p* = 0.015).

**Table 3:** Linear association between V-1P eligibility status and vascular, echocardiographic and biological variables in regression models.

Outcomes	V-1P eligibility	Unadjusted model			Age and sex adjusted		
		Beta	SE	P val	Beta	SE	P val
**Vascular outcomes**							
Intima Media Thickness (µm)	Not eligible	ref			ref		
	PCE	**41**	**17**	**0.016**	12	18	0.51
	PCE & Prevent	**85**	**19**	**<0.001**	**51**	**20**	**0.009**
Pulse Wave Velocity (m/s)	Not eligible	ref			ref		
	PCE	**1.5**	**0.2**	**<0.001**	**0.9**	**0.2**	**<0.001**
	PCE & Prevent	**1.6**	**0.2**	**<0.001**	**0.9**	**0.2**	**<0.001**
Presence of carotid artery plaque, odds ratios (95 % CI)	Not eligible	ref			ref		
	PCE	2.3 (1.3, 3.9)		0.003	1.5 (0.8, 2.7)		0.18
	PCE & Prevent	2.1 (1.2, 3.9)		0.015	OR = 1.3 (0.7, 2.5)		0.41
**Echocardiographic outcomes**		**Beta**	**SE**	**P val**	**Beta**	**SE**	**P val**
Indexed LV Mass (g/m²)	Not eligible	ref			ref		
	PCE	**7.7**	**2.5**	**0.002**	0.1	2.5	0.98
	PCE & Prevent	**8.8**	**2.7**	**0.001**	1.6	2.7	0.55
Indexed LA Volume (mL/m²)	Not eligible	ref			ref		
	PCE	1.1	1.0	0.240	−0.1	1.0	0.92
	PCE & Prevent	0.4	1.1	0.690	−0.9	1.1	0.41
LA reservoir strain, %	Not eligible	ref			ref		
	PCE	−0.4	1.1	0.690	−0.1	1.1	0.95
	PCE & Prevent	**−2.6**	**1.2**	**0.033**	−1.6	1.3	0.20
LV Global Longitudinal Strain	Not eligible	ref			ref		
	PCE	0.7	0.4	0.130	0.3	0.5	0.50
	PCE & Prevent	**2.5**	**0.5**	**<0.001**	**2.3**	**0.5**	**<0.001**
LV ejection fraction %	Not eligible	ref			ref		
	PCE	0.5	0.8	0.560	1.1	0.8	0.19
	PCE & Prevent	−0.6	0.9	0.470	−0.3	0.9	0.71
Indexed LV end-systolic volume	Not eligible	ref			ref		
	PCE	−0.7	0.8	0.370	**−2.3**	**0.8**	**0.004**
	PCE & Prevent	0.1	0.9	0.930	−0.9	0.9	0.29
Indexed LV end-diastolic volume	Not eligible	ref			ref		
	PCE	−0.9	1.6	0.580	**−4.5**	**1.6**	**0.006**
	PCE & Prevent	−1.3	1.8	0.470	**−3.8**	**1.8**	**0.034**
E’ mean, cm/s	Not eligible	ref			ref		
	PCE	**−1.1**	**0.2**	**<0.001**	**−0.9**	**0.3**	**0.001**
	PCE & Prevent	**−1.2**	**0.3**	**<0.001**	**−0.8**	**0.3**	**0.006**
E/A ratio	Not eligible	ref			ref		
	PCE	**−0.1**	**0.0**	**<0.001**	**−0.1**	**0.0**	**0.003**
	PCE & Prevent	**−0.1**	**0.0**	**0.001**	−0.1	0.0	0.06
E/e’ ratio	Not eligible	ref			ref		
	PCE	**0.6**	**0.2**	**0.005**	**0.6**	**0.2**	**0.009**
	PCE & Prevent	**1.4**	**0.2**	**<0.001**	**1.2**	**0.3**	**<0.001**
E wave, m/s	Not eligible	ref			ref		
	PCE	0.0	0.0	0.220	0.0	0.0	0.91
	PCE & Prevent	0.0	0.0	0.190	**0.0**	**0.0**	**0.023**
A wave, m/s	Not eligible	ref			ref		
	PCE	**0.1**	**0.0**	**<0.001**	**0.1**	**0.0**	**<0.001**
	PCE & Prevent	**0.1**	**0.0**	**<0.001**	**0.1**	**0.0**	**<0.001**
Tricuspid annular plane systolic excursion, mm	Not eligible	ref			ref		
	PCE	0.2	0.6	0.750	−0.6	0.6	0.35
	PCE & Prevent	0.3	0.6	0.660	−0.3	0.7	0.61
**Cardiovascular and inflammatory markers**		**Beta**	**SE**	**P val**	**Beta**	**SE**	**P val**
Nt-proBNP*	Not eligible	ref			ref		
	PCE	0.0	0.1	0.960	0.0	0.1	0.83
	PCE & Prevent	**0.3**	**0.1**	**0.009**	0.2	0.1	0.10
Troponin*	Not eligible	ref			ref		
	PCE	**0.3**	**0.1**	**0.013**	0.0	0.1	0.78
	PCE & Prevent	**0.6**	**0.1**	**<0.001**	**0.4**	**0.1**	**0.015**
Interleukin-6*	Not eligible	ref			ref		
	PCE	**0.3**	**0.1**	**0.001**	**0.3**	**0.1**	**0.002**
	PCE & Prevent	**0.7**	**0.1**	**<0.001**	**0.7**	**0.1**	**<0.001**
Growth differentiation factor 15*	Not eligible	ref			ref		
	PCE	**0.3**	**0.1**	**<0.001**	**0.2**	**0.1**	**<0.001**
	PCE & Prevent	**0.5**	**0.1**	**<0.001**	**0.3**	**0.1**	**<0.001**
High Sensitivity C- Reactive Protein, mg/L	Not eligible	ref			ref		
	PCE	**0.5**	**0.1**	**<0.001**	**0.6**	**0.1**	**<0.001**
	PCE & Prevent	**1.0**	**0.1**	**<0.001**	**1.1**	**0.1**	**<0.001**

LA, left atrial; LV left ventricular; PCE pooled cohort equations; PREVENT, Predicting Risk of Cardiovascular Disease Events. *Biomarker concentrations from Olink assays and for hsCRP were log2-transformed to obtain a normal distribution; therefore, a β-coefficient of 1 represents a two-fold higher biomarker concentration.

Both PCE only and PCE+PREVENT eligible participants had more inflammation than ineligible participants. Because Olink-derived biomarkers were analyzed on a log2 scale, regression coefficients can be interpreted as fold-changes, with a β of 1 indicating an approximate doubling of concentration. Using this framework, PCE+PREVENT eligible participants had ∼1.3-fold higher troponin (β=0.4, *p* = 0.015) and ∼1.6-fold higher interleukin-6 (β=0.7, *p* < 0.001) compared with ineligible participants. Both PCE only and PCE+PREVENT groups showed evidence of heightened inflammation, with 1.5 and 2 fold higher levels of hs-CRP (β=0.6 and 1.1, *p* < 0.001 for PCE and PCE+PREVENT, respectively) and 1.2–1.3 fold higher growth differentiation factor-15 levels (β=0.2–0.3, both *p* < 0.001).

## 4 Discussion

In this European cohort of participants initially free from cardiovascular disease, 1 in 6 individuals were V-1P-eligible based on PCE criteria and 1 in 14 based on PREVENT. These findings highlight the generalizability of V-1P results to the general population. V-1P eligible participants—either meeting PCE or PREVENT criteria—had greater subclinical cardiac and vascular abnormalities, elevated inflammatory markers, and higher circulating troponin levels than V-1P ineligible participants, underscoring that V-1P eligible participants may have evidence of early, subclinical cardiovascular disease (Central Illustration). These findings help distinguish PCE and PREVENT equations in a European cohort and suggest that relying solely on PREVENT may miss more than half of potentially at-risk individuals with subclinical disease identified by PCE.

These results complement a prior study in the US which found primary prevention patients who met V-1P eligibility were at high ASCVD risk and had low rates of lipid lowering therapy [20]. Our study extends these findings to a European cohort with lower rates of obesity and greater proportion of women, identifying that many apparently healthy adults were also eligible for V-1P, whether by PCE or PREVENT. The estimated cardiovascular risk of V-1P eligible participants in our cohort is remarkably similar to that reported in the US V-1P eligible population [20]. The 10-year ASCVD risk for V-1P eligible participants in our European cohort was 19 % by PCE and 11 % by PREVENT compared with 21 % by PCE and 12 % by PREVENT in the US, respectively [20]. Given the similarity in estimated ASCVD risk in the two populations, it is likely the observed vascular abnormalities, subclinical cardiac dysfunction, and elevated inflammatory markers might also be evident in U.S. populations meeting V-1P eligibility criteria.

Although prior studies have reported low use of lipid lowering therapy in secondary prevention cohorts, the use of lipid lowering therapy in primary prevention is less well described [21,22]. We found that only 1 in 3 participants who were V-1P eligible were taking statins. Even among people who receive lipid lowering therapy, LDL-C target attainment in Europe is poor [23]. Reasons for inadequate treatment include overuse of low intensity statins or poor adherence to prescribed therapeutic regimens [[23], [24], [25]]. Inclisiran has gained interest for primary prevention because it only requires injections every six months with potential for better adherence whilst maintaining a good efficacy and tolerability profile [7,26,27]. As with any pharmacological treatment, benefits will need to outweigh harms and costs, particularly in a primary prevention cohort where treatment will need to be maintained over years or decades to achieve cumulative benefits [[3], [4], [5]]. Our findings suggest that V-1P eligible participants already exhibit evidence of subclinical cardiovascular disease, inflammation, and low-grade myocardial injury reflected by elevated troponin and these insights may facilitate clinician and patient discussions around such treatment decisions.

When applying V-1P eligibility by PREVENT, fewer patients met V-1P inclusion criteria and these patients were of the highest cardiovascular risk. Use of PCE over PREVENT identified more than twice the number of participants, and these individuals also had evidence of subclinical cardiovascular disease and inflammation compared to V-1P ineligible participants. However, PREVENT is associated with better risk prediction and calibration than PCE, particularly in ethnically diverse populations [28,29]. Nevertheless our findings around V-1P eligibility using PCE are relevant because the V-1P trial started recruitment in 2023 and it is likely that many individuals were enrolled based on PCE rather than PREVENT. Given future cardiovascular prevention guidelines will incorporate the PREVENT equation into clinical decision making for preventive therapies, our findings related to V-1P eligible participants by PREVENT will become increasingly relevant.

Elevated hs-CRP is a predictor of cardiovascular events in the general population without known ASCVD [30]. In our cohort, V-1P eligible participants had higher levels of hs-CRP, and these individuals had evidence of adverse subclinical cardiovascular and inflammatory changes. These findings support the use of hs-CRP as a risk marker in the general population. Similarly, growth differentiation factor 15 was higher in V-1P eligible individuals, and growth differentiation factor 15 is associated with impaired myocardial energetics and function and predicts occurrence of coronary artery disease and incident heart failure [18,19]. In isolation, it is unclear if these increases in biomarkers are clinically significant, but the consistency of these adverse trends across biomarkers and imaging parameters suggests V-1P eligible participants have a higher burden of subclinical cardiovascular injury and may be further along the cardiovascular disease spectrum.

These findings should be interpreted in the context of a few limitations. This study did not assess eligibility for V-1P based on coronary artery calcium score nor evidence of non-obstructive coronary disease on angiography, which were two other entry points into the trial. Therefore, an even greater number of people may be V-1P eligible based on these modalities if they did not meet criteria by calculated ASCVD risk. The cohort was relatively small and from one geographic region, which limits generalizability to broader European populations. We used the STANISLAS cohort because this is a well characterized group of individuals who are free from known ASCVD. This was a cross-sectional study and longitudinal assessment of vascular disease and echocardiographic indices was not available, although the 10-year estimated ASCVD risk equations suggest V-1P eligible participants are at high risk of clinical events.

In this European cohort, a total of 1 in 6 individuals were V-1P-eligible based on 10-year ASCVD risk estimated by PCE and 1 in 14 based on PREVENT, highlighting the high number of seemingly healthy Europeans for whom the V-1P results may apply. Individuals eligible for V-1P—either meeting PCE or PREVENT criteria—showed subclinical cardiac and vascular abnormalities alongside elevated inflammatory markers, underscoring that V-1P criteria may detect early cardiovascular damage. These findings may facilitate shared decision making around long-term pharmacological lipid lowering in V-1P eligible participants who exhibit evidence of subclinical cardiovascular and inflammatory injury.

## Data availability

Data will be made available upon reasonable request by contacting the corresponding author.

## Funding

Nothing to declare.

## Ethical approval

The local ethics committee (Comité de Protection des Personnes Est III, Nancy, France) approved the study protocol and all study participants provided written informed consent in order to participate.

## Pre-registered clinical trial number

Not applicable.

## CRediT authorship contribution statement

**Nelson Wang:** Writing – review & editing, Writing – original draft, Methodology, Formal analysis. **Deepak L. Bhatt:** Writing – review & editing, Writing – original draft, Supervision, Conceptualization. **Constance Xhaard:** Writing – review & editing, Writing – original draft, Methodology, Formal analysis. **Rahul Aggarwal:** Writing – original draft. **Marc P. Bonaca:** Writing – original draft. **Anastasia Lesogor:** Writing – review & editing. **Janna Sand Dejmek:** Writing – review & editing. **Manesh R. Patel:** Writing – review & editing. **Erik S.G. Stroes:** Writing – original draft. **Pam R. Taub:** Writing – review & editing. **Stephan Windecker:** Writing – review & editing. **Kevin Duarte:** Writing – review & editing, Data curation. **Luca Monzo:** Writing – review & editing, Writing – original draft, Data curation. **Guillaume Baudry:** Writing – review & editing, Data curation. **Faiez Zannad:** Writing – review & editing, Supervision, Data curation. **Nicolas Girerd:** Writing – review & editing, Writing – original draft, Visualization, Validation, Supervision, Resources, Project administration, Methodology, Investigation, Funding acquisition, Formal analysis, Data curation, Conceptualization.

## Declaration of competing interest

Dr. Aggarwal is involved in research funded by the Bristol Myers Squibb-Pfizer alliance, Novartis, Lexicon, Cleerly, and Amarin, and is a consultant for Lexicon and Amarin.

Dr. Bhatt discloses the following relationships - Advisory Board: Angiowave, Antlia Bioscience, Bayer, Boehringer Ingelheim, CellProthera, Cereno Scientific, E-Star Biotech, High Enroll, Janssen, Level Ex, McKinsey, Medscape Cardiology, Merck, NirvaMed, Novo Nordisk, Repair Biotechnologies, Stasys, Tourmaline Bio; Board of Directors: American Heart Association New York City, Angiowave (stock options), Bristol Myers Squibb (stock), DRS.LINQ (stock options), High Enroll (stock); Consultant: Alnylam, Altimmune, Broadview Ventures, Corcept Therapeutics, Corsera, GlaxoSmithKline, Hims, SERB, SFJ, Summa Therapeutics, Worldwide Clinical Trials; Data Monitoring Committees: Acesion Pharma, Assistance Publique-Hôpitaux de Paris, Baim Institute for Clinical Research, Boston Scientific (Chair, PEITHO trial), Cleveland Clinic, Contego Medical (Chair, PERFORMANCE 2), Duke Clinical Research Institute, Mayo Clinic, Mount Sinai School of Medicine (for the ABILITY-DM trial, funded by Concept Medical; for ALLAY-HF, funded by Alleviant Medical), Novartis, Population Health Research Institute; Rutgers University (for the NIH-funded MINT Trial); Honoraria: American College of Cardiology (Senior Associate Editor, Clinical Trials and News, ACC.org; Chair, ACC Accreditation Oversight Committee), Arnold and Porter law firm (work related to Sanofi/Bristol-Myers Squibb clopidogrel litigation), Baim Institute for Clinical Research (AEGIS-II executive committee funded by CSL Behring), Belvoir Publications (Editor in Chief, Harvard Heart Letter), Canadian Medical and Surgical Knowledge Translation Research Group (clinical trial steering committees), CSL Behring (AHA lecture), Duke Clinical Research Institute, Engage Health Media, HMP Global (Editor in Chief, Journal of Invasive Cardiology), Medtelligence/ReachMD (CME steering committees), MJH Life Sciences, Oakstone CME (Course Director, Comprehensive Review of Interventional Cardiology), Philips (Becker's Webinar on AI), Population Health Research Institute, WebMD (CME steering committees), Wiley (steering committee); Other: Clinical Cardiology (Deputy Editor); Progress in Cardiovascular Diseases (Deputy Editor); Patent: Sotagliflozin (named on a patent for sotagliflozin assigned to Brigham and Women's Hospital who assigned to Lexicon; neither I nor Brigham and Women's Hospital receive any income from this patent); Research Funding: Abbott, Acesion Pharma, Afimmune, Alnylam, Amarin, Amgen, AstraZeneca, Atricure, Bayer, Boehringer Ingelheim, Boston Scientific, CellProthera, Cereno Scientific, Chiesi, Cleerly, CSL Behring, Faraday Pharmaceuticals, Fractyl, Idorsia, Janssen, Javelin, Lexicon, Lilly, Medtronic, Merck, MiRUS, Moderna, Novartis, Novo Nordisk, Pfizer, PhaseBio, Regeneron, Reid Hoffman Foundation, Roche, Sanofi, Stasys, 89Bio; Royalties: Elsevier (Editor, Braunwald’s Heart Disease); Site Co-Investigator: Cleerly.

Dr. Bonaca is the Executive Director of CPC, a non-profit academic research organization affiliated with the University of Colorado, that receives or has received research grant/consulting funding between August 2021 and present from: Abbott Laboratories, Agios Pharmaceuticals, Inc., Alexion Pharma, Alnylam Pharmaceuticals, Inc., Amgen Inc., Angionetics, Inc., Anthos Therapeutics, Array BioPharma, Inc., AstraZeneca and Affiliates, Atentiv LLC, Audentes Therapeutics, Inc., Bayer and Affiliates, Bristol-Meyers Squibb Company, Cambrian Biopharma, Inc., Cardiol Therapeutics Inc., CellResearch Corp., Cleerly Inc., Cook Regentec LLC, CSL Behring LLC, Eidos Therapeutics, Inc., EP Trading Co. Ltd., Epizon Pharma, Inc., Esperion Therapeutics, Inc., Everly Well, Inc., Exicon Consulting Pvt. Ltd., Faraday Pharmaceuticals, Inc., Foresee Pharmaceuticals Co. Ltd., Fortress Biotech, Inc., HDL Therapeutics Inc., HeartFlow Inc., Hummingbird Bioscience, Insmed Inc., Ionis Pharmaceuticals, Janssen and Affiliates, Kowa Research Institute, Inc., Lexicon Pharmaceuticals, Inc., Medimmune Ltd., Merck & Affiliates, Nectero Medical Inc., Novartis Pharmaceuticals Corp., Novo Nordisk, Inc., Osiris Therapeutics Inc., Pfizer Inc., PhaseBio Pharmaceuticals, Inc., Prairie Education and Research Cooperative, Prothena Biosciences Limited, Regeneron Pharmaceuticals, Inc., Regio Biosciences, Inc., Sanofi-Aventis Groupe, Silence Therapeutics PLC, Smith & Nephew plc, Stealth BioTherapeutics Inc., VarmX, Virta Health Corporation.

Dr. Lesogor is an employee of Novartis Pharma AG, Switzerland.

Dr. Sand Dejmek is an employee of Novartis Pharma AG, Switzerland.

Dr. Patel reports research grants with Novartis, Bayer, Jansen, Idorsia, NHBLI. Dr. Patel has advisory/consulting relationships with Esperion, Bayer, Jansen, and Idorsia.

Dr. Stroes has received adboard/consultancy fees paid to institution by Amgen, Novartis, Novo-Nordisk, Merck, Ionis, Astra-Zeneca and Ultragenyx.Dr. Taub reports a relationship with Novartis, Esperion Therapeutics Inc., Amarin, Amgen Inc., Novo Nordisk Inc., Medtronic Inc., Edwards, Inc., Boehringer Ingelheim, Jazz Pharmaceuticals, Milestone Pharmaceuticals, Bayer, and Lilly that includes consulting or advisory role. Dr. Taub serves as an advisory board member and/or member of the steering/executive committee of trials funded by Novartis, Merck, Amgen, Arrowhead, Cleerly, Medtronic Boehringer Ingelheim, CSL Behring and Lilly.

Dr. Windecker reports research, travel or educational grants to the institution without impact on his personal remuneration from Abbott, Abiomed, Amgen, Astra Zeneca, Bayer, Bbraun, Biotronik, Boehringer Ingelheim, Boston Scientific, Bristol Myers Squibb, Cardinal Health, CardioValve, Cleerly Inc., Cordis Medical, Corflow Therapeutics, CSL Behring, Daiichi Sankyo, Edwards Lifesciences, Farapulse Inc. Fumedica, GE Medical Systems, Gebro Pharma, Guerbet, Idorsia, Inari Medical, InfraRedx, Janssen-Cilag, Johnson & Johnson, Medalliance, Medicure, Medtronic, Merck Sharp & Dohm, Miracor Medical, Neucomed, Novartis, Novo Nordisk, Organon, OrPha Suisse, Pharming Tech, Pfizer, Philips AG, Polares, Regeneron, Sanofi-Aventis, Servier, Siemens Healthcare, Sinomed, SMT Sahajanand Medical Technologies, Terumo, Vifor, V-Wave, Zoll Medical. Dr. Windecker reports research, travel and/or educational grants to the institution from Abbott, Abiomed, Alnylam, Amicus Therapeutics, Amgen, Anteris, Astra Zeneca, Bayer, B.Braun, Bioanalytica, Biotronik, Boehringer Ingelheim, Boston Scientific, Bristol Myers Squibb, Cordis Medical, CorFlow Therapeutics, CSL Behring, Daiichi Sankyo, Edwards Lifesciences, Fumedica, GE Healthcare, Guerbet, IACULIS, Inari Medical, Janssen AI, Johnson & Johnson, Medalliance, Medtronic, MSD Merck Sharp & Dohme, Neovii Pharmaceutica, Neutromedics AG, Novartis, Novo Nordisk, OM Pharma, Optimapharm, Orchestra BioMed, Pfizer, Philips AG, Sanofi-Aventis, Servier, Shockwave Medical, Siemens Healthcare, Sinomed, SMT Sahajanand Medical Technologies, Vascular Medical, V-Wave. Dr. Windecker serves as advisory board member and/or member of the steering/executive group of trials funded by Abbott, Amgen, Anteris, Abiomed, Edwards Lifesciences, EnCarda Inc., Medtronic, Novartis, Sinomed with payments to the institution but no personal payments. He is also member of the steering/executive committee group of several investigator-initiated trials that receive funding by industry without impact on his personal remuneration.

Dr. Monzo received a travel grant from Boehringer

G.B. has received personal fees from Abbott, AstraZeneca, Boehringer Ingelheim, Novartis and Novo Nordisk.

Dr Zannad has received personal fees from 89Bio, Abbott, Acceleron, Applied Therapeutics, Bayer, Betagenon, Boehringer, BMS, CVRx, Cambrian, Cardior, Cereno pharmaceutical, Cellprothera, CEVA, Inventiva, KBP, Merck, Novo Nordisk, Owkin, Otsuka, Roche Diagnostics, Northsea, and Us2.ai; has stock options in G3Pharmaceutical and holds equities in Cereno, Cardiorenal, and Eshmoun Clinical Research; and is the founder of Cardiovascular Clinical Trialists.

Dr. Girerd reports honoraria from AstraZeneca, Bayer, Boehringer, Cardiostory, Echosens, GSK, Lilly, NP Medical, Novartis, Novo Nordisk.

Other authors did not report links with industry.
